# Coherent control of quasi-degenerate stationary-like states via multiple resonances

**DOI:** 10.1038/s41598-017-00041-x

**Published:** 2017-02-02

**Authors:** Yunrong Luo, Kuo Hai, Mingliang Zou, Wenhua Hai

**Affiliations:** 1 Department of Physics, and Key Laboratory of Low Dimensional Quantum Structures and Quantum Control of Ministry of Education, Hunan Normal University, Changsha, 410081 China; 2 Synergetic Innovation Center for Quantum Effects and Applications, Hunan Normal University, Changsha, 410081 China

## Abstract

We use three bosons held in a depth-tilt combined-modulated double-well to study coherent control of quantum transitions between *quasi-degenerate stationary-like states* (QDSLSs) with the same quasienergy. Within the high-frequency approximation and for multiple-resonance conditions, we analytically obtain the different QDSLSs including the maximal bipartite entangled states, which enable us to manipulate the transitions between QDSLSs without the observable multiphoton absorption and to simulate a two-qubit system with the considered bosons. The analytical results are confirmed numerically and good agreement is shown. The quantum transitions between QDSLSs can be observed and controlled by adjusting the initial and the final atomic distributions in the currently proposed experimental setup, and possess potential applications in qubit control based on the bipartite entangled states and in engineering quantum dynamics for quantum information processing.

## Introduction

Quantum transition between stationary states plays a crucial role in much of quantum mechanics, while the coherent control of quantum transition is a master key for its practical applications such as the quantum information processing^1^, precision measurement^2^ and so on. Recently, there have been many works focusing on the coherent control of quantum transition in both theoretical and experimental sides^3–13^. Usually, the stimulated quantum transitions between quantum states associated with different energies can be controlled by using photon resonance. In a single-frequency driven system, the notion of resonance transition was associated with the driving frequency fitting a level difference between two internal electronic states^14^ or two external motional states^15–19^. Quantum states also can transit spontaneously from a high level to a low level. The spontaneous quantum transition is one of the reasons for producing the decoherence in quantum information processing. A double-well trapped many-boson system or an atomic Bose-Einstein condensates (BEC) in two different hyperfine states trapped in a single trap can be treated as a bipartite system of two modes^19–21^. The bipartite entangled states in such a system have been investigated, which can be used to encode the qubit^22^. As a maximal bipartite entangled state the NOON state has also been widely studied^22,23^. In order to eliminate the adverse decoherence arising from the spontaneous transitions, we hope to seek the quasi-degenerate bipartite states associated with the same energy^24^ and with the different external fields, and to control the transitions between them by adjusting the field parameters.

The optically trapped atoms offer robust quantum coherence and controllability, providing an attractive system for quantum information processing and for the simulation of complex physical problems^25,26^. When a depth-tilt combined-modulated double-well atomic system with two different driving frequencies are considered, the resonance between driving frequencies becomes possible. The multiple resonances which contain several different forms of resonances will bring new applicable effects for the quantum control^27–30^. In a system of double-well trapped few particles, any one of the above-mentioned motional states corresponds to a certain atomic distribution, which can be expanded in terms of Fock basis. If the probability amplitude of the system being in any Fock state is time-dependent and the corresponding probability is a constant, we call the quantum state the stationary-like (or quasistationary) state (SLS)^31,32^. A SLS with invariant population may be a single Floquet state or a superposition of Floquet states. Quantum transition between different SLSs is as important as that between usual stationary states. When the SLSs have the same Floquet quasienergy, the transitions between them cannot be directly related to the observable multiphoton absorption^15,16^. We define such SLSs as *quasi-degenerate stationary-like states* (QDSLSs), including the CDT (coherent destruction of tunneling) single state with only a single Fock basis^33^, and the NOON state (a superposition of *N* particles in well 1 with zero particle in well 2 and vice versa)^30^. We will demonstrated that the QDSLSs may be prepared by using the multiple-resonance effects. Then we define SCDT (selective coherent destruction of tunneling) state as a superposition of *n* Fock states with time-dependent occupied probabilities, where *n* is less than the dimension of the considered Hilbert space^33^. In such a state, transition of the system to any one of the lacked Fock states can be suppressed selectively. While any pair of QDSLSs can be coherently connected via SCDT states by changing the corresponding system parameters. In recent experiments of Bloch group^6^, high controllability of numbers of atoms within a given well has been realized. Therefore, it is feasible to experimentally control quantum transitions between QDSLSs of different atomic distributions.

In this paper, we consider three bosons held in a depth-tilt combined-modulated double-well and propose a new formalism to study coherent control of quantum transitions between QDSLSs without quasienergy difference. Within the high-frequency approximation and for multiple-resonance conditions, we analytically obtain all Floquet eigenstates and quasienergies. When the driving parameters and initial conditions are adjusted appropriately, a superposition state of Floquet states becomes one of the QDSLSs or a SCDT state^33–35^ that means the transitions between some states are suppressed selectively and the Rabi oscillations between the other QDSLSs occur. Thus the coherent control of quantum transitions between QDSLSs with the same Floquet quasienergy can be realized transparently via the analytical solutions. The results are confirmed numerically and good agreements are found. The transitions between the QDSLSs without quasi-level difference are equivalent to the related population transfers, and can be observed and controlled by adjusting the corresponding atomic distributions with the current experimental capability^5,6^. The results may be used to simulate a two-qubit system with the considered bosons, which is useful in performing the two-qubit logical operations^25,26^ for quantum information processing.

## Results

### Analytical solutions in the high-frequency approximation

We consider three bosons held in a driven and tilted double well potential which can be generated experimentally from a train of optical double wells in the form refs 5 and 36

1 $$\begin{matrix}V(x,t) & = & {V}_{dw}(x,t)+{V}_{tilt}(x,t),\\ {V}_{dw} & = & {V}_{1}{\cos }^{2}x+{V}_{2}{\cos }^{2}[x\,\cos (\frac{\pi }{3}+\epsilon \,\cos (\omega ^{\prime} t))],\\ {V}_{tilt} & = & [{F}_{0}+{F}_{1}\,\cos (\omega t)]x\end{matrix}$$

for the spatial range of a single double well. Here *F*_0,1_ denote the force constants, *V*_1,2_ are the amplitudes of the two optical potentials forming the double-well structure^5^ and $\epsilon$ a small dimensionless constant describing the deviation from the incidence angle of (*π*/3), and (*ω*, *ω*′) the driving frequencies. Experimentally, the optical double well *V*_*dw*_(*x*, *t*) can be formed by two driven laser standing waves^5,6^, and the tilt potential *V*_*tilt*_(*x*, *t*) can be produced by a magnetic field gradient^7,8^ or a periodic shaking of the optical lattice^9^. In Eq. (1), the potentials have been normalized in units of the recoil energy ${E}_{r}=\frac{{\hslash }^{2}{k}^{2}}{2m}$ of atom with mass *m*; the position *x*, driving frequencies (*ω*, *ω*′) and time *t* are in units of the inverse wave vector *k* ^−1^, recoil frequency *ω*_*r*_ = *E*_*r*_/*ħ* and inverse recoil frequency ${\omega }_{r}^{-1}$, respectively. Thus the amplitudes *V*_1,2_ and force constants *F*_0,1_ are in units of *E*_*r*_ and *kE*_*r*_. For the ultracold ^87^Rb atoms the typical *ω*_*r*_ value is about 5 × 10^3^ Hz in the experiment^37^.

In the two-mode approximation^38,39^, the Hamiltonian governing this system reads^30,40^

2 $$\hat{H}(t)=-\,{\rm{\Omega }}(t)({\hat{c}}_{1}^{\dagger }{\hat{c}}_{2}+{\hat{c}}_{2}^{\dagger }{\hat{c}}_{1})+\varepsilon (t)({\hat{c}}_{2}^{\dagger }{\hat{c}}_{2}-{\hat{c}}_{1}^{\dagger }{\hat{c}}_{1})+\frac{U}{2}({\hat{c}}_{1}^{\dagger }{\hat{c}}_{1}^{\dagger }{\hat{c}}_{1}{\hat{c}}_{1}+{\hat{c}}_{2}^{\dagger }{\hat{c}}_{2}^{\dagger }{\hat{c}}_{2}{\hat{c}}_{2}),$$

where ${\hat{c}}_{\mathrm{1,2}}^{\dagger }$ $({\hat{c}}_{1,2})$ denote atomic creation (annihilation) operators in the well 1 and 2, respectively, $\hat{H}$, Ω, *ε* and *U* have been normalized in units of *E*_*r*_. The coupling parameter Ω is given by the integral^36,41,42^

3 $$\begin{matrix}{\rm{\Omega }}(t) & = & {\int }_{-\infty }^{+\infty }dx{w}^{\ast }(x-{x}_{1})[-\,{\nabla }^{2}+V(x,t)]w(x-{x}_{2})\\  & = & {{\rm{\Omega }}}_{0}+{{\rm{\Omega }}}_{1}\,\cos (\omega ^{\prime} t).\end{matrix}$$

Here, *w*(*x* − *x*_*q*_) (*q* = 1, 2) are the dimensionless Wannier states in units of *k* ^−1/2^ and *x*_1,2_ are two positions of the minimal potential^36^. Generally, the coupling parameters obey the inequality^30^ 0 ≤ Ω_1_ ≤ Ω_0_. For a fixing *V*_1_ the values of Ω_0_ and Ω_1_ increase with the increase of *V*_2_ and $|\epsilon |$, respectively. The time-dependent bias *ε*(*t*) is related to the linear potential^36^,

4 $$\begin{matrix}\varepsilon (t) & = & [{F}_{0}+{F}_{1}\,\cos (\omega t)]{\int }_{-\infty }^{+\infty }dx{w}^{\ast }(x-{x}_{q})xw(x-{x}_{q})\\  & = & {\varepsilon }_{0}+{\varepsilon }_{1}\,\cos (\omega t).\end{matrix}$$

Here parameters *ε*_0,1_ are proportional to the force constants *F*_0,1_ respectively, which are two key adjustable parameters for our control schemes. The on-site interaction intensity is in the form

5 $$U=\frac{4\pi {a}_{1D}}{m}{\int }_{-\infty }^{+\infty }dx{w}^{4}(x-{x}_{q})$$

with *a*_1*D*_ being the renormalized *s*-wave scattering length in one-dimensional case^43^, which can be adjusted experimentally in a wide range by the Feshbach-resonance technique^44^.

Using the Fock basis |*i*〉 = |*i*, 3 − *i*〉 with *i* atom(s) being in the left well and 3 − *i* atom(s) being in the right well, we expand the quantum state |*ψ*(*t*)〉 of three-body system (2) as

6 $$|\psi (t)\rangle =\sum _{i=0}^{3}{a}_{i}(t)|i,3-i\rangle $$

in the four dimensional Hilbert space. Here *a*_*i*_(*t*) for *i* = 0, 1, 2, 3 denote the time-dependent probability amplitudes with *i* atom(s) bing in the left well, which obey the normalization condition ${{\rm{\Sigma }}}_{i=0}^{3}{|{a}_{i}(t)|}^{2}=1$. Inserting Eqs (2) and (6) into the Schrödinger equation $i\frac{\partial \psi (t)}{\partial t}=\hat{H}(t)\psi (t)$ results in the coupled equations

7 $$\begin{matrix}i{\dot{a}}_{0}(t) & = & -\,\sqrt{3}{\rm{\Omega }}(t){a}_{1}(t)+[3U+3\varepsilon (t)]{a}_{0}(t),\\ i{\dot{a}}_{1}(t) & = & -\,\sqrt{3}{\rm{\Omega }}(t){a}_{0}(t)-2{\rm{\Omega }}(t){a}_{2}(t)+[U+\varepsilon (t)]{a}_{1}(t),\\ i{\dot{a}}_{2}(t) & = & -\,2{\rm{\Omega }}(t){a}_{1}(t)-\sqrt{3}{\rm{\Omega }}(t){a}_{3}(t)+[U-\varepsilon (t)]{a}_{2}(t),\\ i{\dot{a}}_{3}(t) & = & -\,\sqrt{3}{\rm{\Omega }}(t){a}_{2}(t)+[3U-3\varepsilon (t)]{a}_{3}(t).\end{matrix}$$

It is difficult to obtain the exact solutions of Eq. (7), because of the periodically varying coefficients. However, in the high-frequency approximation, it can become a set of linear equations with constant coefficients, which is analytically solvable. To do so, we employ the *multiple-resonance conditions U* = *nω*, *ω*′ = *mω*, *ε*_0_ = *lω* with *n*, *m*, *l* being integers. Any one of the conditions implies a particular resonant mechanism and can cause different tunneling effect^27–30^. We desire that their combination will result in multiple-resonance effects which can be applied to manipulate the system, although the corresponding control protocol may be constrained partially.

In high-frequency case, we introduce the slowly varying functions *b*_*i*_(*t*) through the transformations *a*_0_(*t*) = *b*_0_(*t*)*e* ^−*i*∫[3*U*+3*ε*(*t*)]*dt*^, *a*_1_(*t*) = *b*_1_(*t*)*e* ^−*i*∫[*U*+*ε*(*t*)]*dt*^, *a*_2_(*t*) = *b*_2_(*t*)*e* ^−*i*∫[*U*−*ε*(*t*)]*dt*^, *a*_3_(*t*) = *b*_3_(*t*)*e* ^−*i*∫[3*U*−3*ε*(*t*)]*dt*^, and use the Fourier expansion $\exp [\pm i\int \varepsilon (t)dt]={\sum }_{{n}^{^{\prime} }=-\infty }^{\infty }{J}_{{n}^{^{\prime} }}(\pm \frac{{\varepsilon }_{1}}{\omega })\exp [i(n^{\prime} \pm l)\omega t]$ with ${J}_{n^{\prime} }(\frac{{\varepsilon }_{1}}{\omega })$ being the *n*′-order Bessel function of $\frac{{\varepsilon }_{1}}{\omega }$ and obeying ${J}_{n^{\prime} }(-\frac{{\varepsilon }_{1}}{\omega })={J}_{-n^{\prime} }(\frac{{\varepsilon }_{1}}{\omega })$. Noticing $U=n\omega ,{\rm{\Omega }}(t)={{\rm{\Omega }}}_{0}+\frac{1}{2}{{\rm{\Omega }}}_{1}({e}^{im\omega t}+{e}^{-im\omega t})$ and neglecting the rapidly oscillating terms with *n*′ + 2*l* ≠ 0, *n*′ ± 2*n* + 2*l* ≠ 0, *n*′ + 2*l* ± *m* ≠ 0 and *n*′ ± 2*n* + 2*l* ± *m* ≠ 0, Eq. (7) is transformed to the form

8 $$\begin{matrix}i{\dot{b}}_{0}(t) & = & -\,\frac{\sqrt{3}}{2}{\eta }_{1}{b}_{1}(t),\\ i{\dot{b}}_{1}(t) & = & -\,\frac{\sqrt{3}}{2}{\eta }_{1}{b}_{0}(t)-{\eta }_{2}{b}_{2}(t),\\ i{\dot{b}}_{2}(t) & = & -\,{\eta }_{2}{b}_{1}(t)-\frac{\sqrt{3}}{2}{\eta }_{3}{b}_{3}(t),\\ i{\dot{b}}_{3}(t) & = & -\,\frac{\sqrt{3}}{2}{\eta }_{3}{b}_{2}(t),\end{matrix}$$

where the coupling constants have been renormalized as

9 $$\begin{matrix}{\eta }_{1} & = & 2{{\rm{\Omega }}}_{0}{J}_{-2n-2l}(\frac{2{\varepsilon }_{1}}{\omega })+{{\rm{\Omega }}}_{1}[{J}_{-2n-2l-m}(\frac{2{\varepsilon }_{1}}{\omega })+{J}_{-2n-2l+m}(\frac{2{\varepsilon }_{1}}{\omega })],\\ {\eta }_{2} & = & 2{{\rm{\Omega }}}_{0}{J}_{-2l}(\frac{2{\varepsilon }_{1}}{\omega })+{{\rm{\Omega }}}_{1}[{J}_{-2l-m}(\frac{2{\varepsilon }_{1}}{\omega })+{J}_{-2l+m}(\frac{2{\varepsilon }_{1}}{\omega })],\\ {\eta }_{3} & = & 2{{\rm{\Omega }}}_{0}{J}_{2n-2l}(\frac{2{\varepsilon }_{1}}{\omega })+{{\rm{\Omega }}}_{1}[{J}_{2n-2l-m}(\frac{2{\varepsilon }_{1}}{\omega })+{J}_{2n-2l+m}(\frac{2{\varepsilon }_{1}}{\omega })].\end{matrix}$$

**Figure 1:**
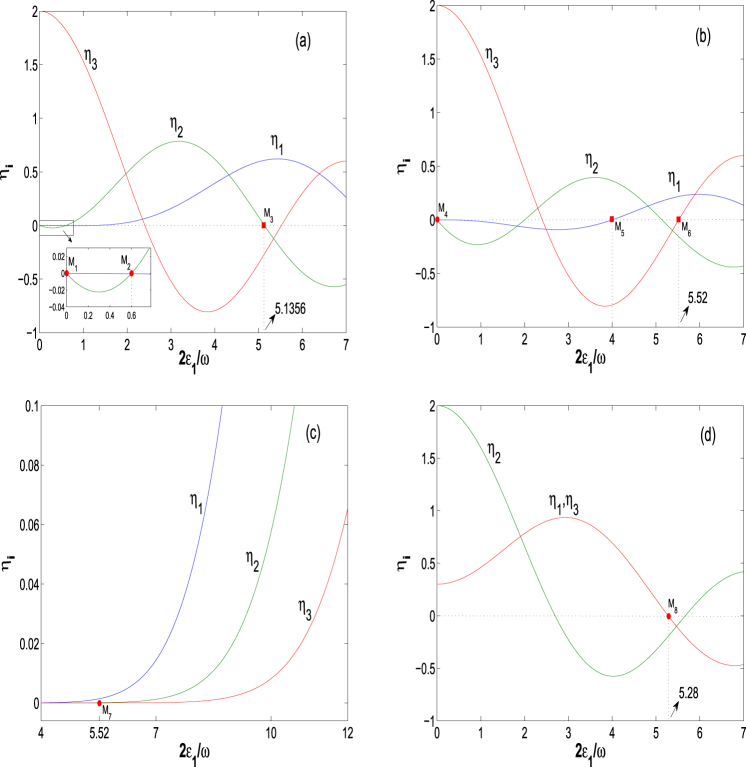
The renormalized coupling coefficients *η*_*i*_ as functions of the driving parameter 2*ε*_1_/*ω* for *ω* = 20, Ω_0_ = 1, *n* = 1 and (**a**) *m* = *l* = 1, Ω_1_ = 0.3; (**b**) *m* = *l* = 1, Ω_1_ = 1; (**c**) *m* = 1, *l* = −7, Ω_1_ = 1; (**d**) *m* = 2, *l* = 0, Ω_1_ = 0.3. By the points we mean the zero-points *M*_*i*_ = *M*_*i*_(2*ε*_1_/*ω*, *η*_1_, *η*_2_, *η*_3_) of part *η*_*j*_. Hereafter, any parameter adopted in the figures is dimensionless.

The renormalized effective coupling coefficients *η*_*j*_ directly determine the solutions of Eq. (8), which are adjusted by the system parameters. At any zero-point of *η*_*j*_, Eq. (8) will be partly decoupled and its solutions will be consequently simplified. In Fig. 1, we plot the *η*_*i*_ as functions of the driving parameter 2*ε*_1_/*ω* for *ω* = 20, Ω_0_ = 1, *n* = 1 (*U* = *ω*) and (a) *m* = *l* = 1 (*ω*′ = *ε*_0_ = *ω*), Ω_1_ = 0.3; (b) *m* = *l* = 1 (*ω*′ = *ε*_0_ = *ω*), Ω_1_ = 1; (c) *m* = 1 (*ω*′ = *ω*), *l* = −7 (*ε*_0_ = −7*ω*), Ω_1_ = 1; (d) *m* = 2 (*ω*′ = 2*ω*), *l* = 0 (*ε*_0_ = 0), Ω_1_ = 0.3. Expressing the zero-points of part *η*_*j*_ as *M*_*i*_ = *M*_*i*_(2*ε*_1_/*ω*, *η*_1_, *η*_2_, *η*_3_), we label eight zero-points in Fig. 1 with the inset as *M*_1_ = *M*_1_(0, 0, 0, 2), *M*_2_ = *M*_2_(0.6, 0, 0, 1.82), *M*_3_ = *M*_3_(5.1356, 0.61, 0, −0.26) for the other parameters of Fig. 1(a); *M*_4_ = *M*_4_(0, 0, 0, 2), *M*_5_ = *M*_5_(4, 0, 0.36, −0.79), *M*_6_ = *M*_6_(5.52, 0.22, −0.16, 0) for the other parameters of Fig. 1(b); *M*_7_ = *M*_7_(5.52, 0.001, 0, 0) and *M*_8_ = *M*_8_(5.28, 0, −0.19, 0) for the other parameters of Fig. 1(c) and (d) respectively. The parameters associated with these zero-points are related to the collapse points of quasienergy spectrum and the CDT^13^, which will be adopted in our control proposals of quantum transitions.

### Quasienergies and Floquet states

According to the Floquet theorem^30,45,46^, Eq. (6) contains the Floquet solution |*ψ*(*t*)〉 = |*φ*(*t*)〉*e* ^−*iEt*^, where *E* is called the quasienergy and |*φ*(*t*)〉 = |*φ*(*t* + 2*π*/*ω*)〉 is the corresponding Floquet state. Noticing the multiple resonance conditions and the relations between *a*_*i*_(*t*) and *b*_*i*_(*t*), the Floquet state can be represented as |*φ*(*t*)〉 = *Ae* ^−*i*∫(3*U*+3*ε*(*t*))*dt*^|0, 3〉 + *Be* ^−*i*∫(*U*+*ε*(*t*))*dt*^|1, 2〉 + *Ce* ^−*i*∫(*U*−*ε*(*t*))*dt*^|2, 1〉 + *De* ^−*i*∫(3*U*−3*ε*(*t*))*dt*^|3, 0〉, through the stationary solutions of Eq. (8), *b*_0_(*t*) = *Ae* ^−*iEt*^, *b*_1_(*t*) = *Be* ^−*iEt*^, *b*_2_(*t*) = *Ce* ^−*iEt*^, *b*_3_(*t*) = *De* ^−*iEt*^, where *A*, *B*, *C*, *D* are constant amplitudes satisfying the normalization condition |*A*|^2^ + |*B*|^2^ + |*C*|^2^ + |*D*|^2^ = 1, and the slowly varying functions *b*_*i*_(*t*) require the quasienergy to satisfy |*E*| ≪ *ω*. Inserting the stationary solutions into Eq. (8), one obtain the four Floquet quasienergies *E*_*j*_ and four sets of the constant amplitudes *A*_*j*_, *B*_*j*_, *C*_*j*_, *D*_*j*_ (*j* = 1, 2, 3, 4) as

10 $$\begin{matrix}{E}_{1} & = & -\,{E}_{2}=\frac{{\rho }_{+}}{2\sqrt{2}},{E}_{3}=-\,{E}_{4}=\frac{{\sigma }_{+}}{2\sqrt{2}},\\ {C}_{1} & = & {C}_{2}=\frac{12\sqrt{2}}{\sqrt{288(1+\frac{6{\eta }_{3}^{2}}{{\rho }_{+}^{2}})+\frac{{\rho }_{-}^{4}(6{\eta }_{1}^{2}+{\rho }_{+}^{2})}{{\eta }_{1}^{4}{\eta }_{2}^{2}}}},\\ {C}_{3} & = & {C}_{4}=\frac{12\sqrt{2}}{\sqrt{288(1+\frac{6{\eta }_{3}^{2}}{{\sigma }_{+}^{2}})+\frac{{\sigma }_{-}^{4}(6{\eta }_{1}^{2}+{\sigma }_{+}^{2})}{{\eta }_{1}^{4}{\eta }_{2}^{2}}}},\\ {A}_{1} & = & {A}_{2}=-\,\frac{{\rho }_{-}^{2}{C}_{1}}{4\sqrt{3}{\eta }_{1}{\eta }_{2}},{A}_{3}={A}_{4}=\frac{{\sigma }_{-}^{2}{C}_{3}}{4\sqrt{3}{\eta }_{1}{\eta }_{2}},\\ {B}_{1} & = & -\,{B}_{2}=\frac{{\rho }_{-}^{2}{\rho }_{+}{C}_{1}}{12\sqrt{2}{\eta }_{1}^{2}{\eta }_{2}},{B}_{3}=-\,{B}_{4}=-\,\frac{{\sigma }_{-}^{2}{\sigma }_{+}{C}_{3}}{12\sqrt{2}{\eta }_{1}^{2}{\eta }_{2}},\\ {D}_{1} & = & -\,{D}_{2}=-\,\frac{\sqrt{6}{\eta }_{3}{C}_{1}}{{\rho }_{+}},{D}_{3}=-\,{D}_{4}=-\,\frac{\sqrt{6}{\eta }_{3}{C}_{3}}{{\sigma }_{+}}.\end{matrix}$$

Here, some new constants are adopted as ${\sigma }_{\pm }=\sqrt{3{\eta }_{1}^{2}+\sqrt{{(3{\eta }_{1}^{2}+4{\eta }_{2}^{2}+3{\eta }_{3}^{2})}^{2}-36{\eta }_{1}^{2}{\eta }_{3}^{2}}\pm (4{\eta }_{2}^{2}+3{\eta }_{3}^{2})}$, ${\rho }_{\pm }=\sqrt{4{\eta }_{2}^{2}+3{\eta }_{3}^{2}\pm (3{\eta }_{1}^{2}-\sqrt{{(3{\eta }_{1}^{2}+4{\eta }_{2}^{2}+3{\eta }_{3}^{2})}^{2}-36{\eta }_{1}^{2}{\eta }_{3}^{2}})}$. Because the renormalized coupling constants *η*_*j*_ in Eq. (9) are adjusted by the system parameters, all the quasienergies *E*_*j*_ and amplitudes *A*_*j*_, *B*_*j*_, *C*_*j*_, *D*_*j*_ are determined by a set of fixed parameters. From Eq. (10), we immediately obtain the four *Floquet states*

11 $$\begin{matrix}\left|{\varphi }_{j}(t)\right\rangle  & = & {A}_{j}{e}^{-3i[(n+l)\omega t+\frac{{\varepsilon }_{1}}{\omega }\sin (\omega t)]}\left|0,3\right\rangle +{B}_{j}{e}^{-i[(n+l)\omega t+\frac{{\varepsilon }_{1}}{\omega }\sin (\omega t)]}\left|1,2\right\rangle \\  &  & +{C}_{j}{e}^{-i[(n-l)\omega t-\frac{{\varepsilon }_{1}}{\omega }\sin (\omega t)]}\left|2,1\right\rangle +{D}_{j}{e}^{-3i[(n-l)\omega t-\frac{{\varepsilon }_{1}}{\omega }\sin (\omega t)]}\left|3,0\right\rangle \end{matrix}$$

for *j* = 1, 2, 3, 4.

It is known that these Floquet states are the SLSs with variable probability amplitudes and invariant populations^31,32^. Quantum population transfer of any particle cannot occur in a single Floquet state $|{\psi }_{j}(t)\rangle =|{\varphi }_{j}(t)\rangle {e}^{-i{E}_{j}t}$, but it can occur in a linear superposition state of the Floquet states. The system may occupy a single Floquet state only for some fixed initial conditions and system parameters.

### General coherent superposition state

In order to study population transfer of the system, we have to consider the coherent superposition of the Floquet states. According to the superposition principle of quantum mechanics, linear superposition of the Floquet states which constitute a set of complete bases^36,47^ is still a solution of the Schrödinger equation. Directly employing Eqs (10) and (11) to the linear superposition yields the general superposition state

12 $$\begin{matrix}\left|\psi (t)\right\rangle  & = & \sum _{j=1}^{4}{s}_{j}\left|{\varphi }_{j}(t)\right\rangle {e}^{-i{E}_{j}t}\\  & = & {b}_{0}^{^{\prime} }(t){e}^{-3i[(n+l)\omega t+\frac{{\varepsilon }_{1}}{\omega }\sin (\omega t)]}\left|0,3\right\rangle +{b}_{1}^{^{\prime} }(t){e}^{-i[(n+l)\omega t+\frac{{\varepsilon }_{1}}{\omega }\sin (\omega t)]}\left|1,2\right\rangle \\  &  & +{b}_{2}^{^{\prime} }(t){e}^{-i[(n-l)\omega t-\frac{{\varepsilon }_{1}}{\omega }\sin (\omega t)]}\left|2,1\right\rangle +{b}_{3}^{^{\prime} }(t){e}^{-3i[(n-l)\omega t-\frac{{\varepsilon }_{1}}{\omega }\sin (\omega t)]}\left|3,0\right\rangle ,\end{matrix}$$

where *s*_*j*_ are superposition coefficients adjusted by the initial conditions and normalization, and the probability amplitudes are renormalized as ${b}_{0}^{^{\prime} }(t)={\sum }_{j=1}^{4}{s}_{j}{A}_{j}{e}^{-i{E}_{j}t},{b}_{1}^{^{\prime} }(t)={\sum }_{j=1}^{4}{s}_{j}{B}_{j}{e}^{-i{E}_{j}t}$, ${b}_{2}^{^{\prime} }(t)={\sum }_{j=1}^{4}{s}_{j}{C}_{j}{e}^{-i{E}_{j}t},{b}_{3}^{^{\prime} }(t)={\sum }_{j=1}^{4}{s}_{j}{D}_{j}{e}^{-i{E}_{j}t}$ with constants *E*_*j*_, *A*_*j*_, *B*_*j*_, *C*_*j*_, *D*_*j*_ being given in Eq. (10). The occupy probabilities of *i* bosons in left well read as ${P}_{i}={|{b}_{i}^{^{\prime} }(t)|}^{2}$ for *i* = 0, 1, 2, 3. The first line of Eq. (12) means that the general superposition state can be reduced to a new Floquet state, if and only if quasienergy of every nonzero term take the same value. Generally, the superposition state (12) does not satisfy the definition of the Flouqet state. The coherent superposition implies quantum interference effect among the four Floquet states with different quasienergies. It may cause the coherent enhancement or suppression of quantum tunneling with adjustable degree by changing the driving parameters^48^. Such an interference effect will be applied to manipulate the quantum transfer between QDSLSs.

### Coherent control of quantum transitions between QDSLSs

Under the multiple-resonance conditions and high-frequency approximation, we have obtained the general form (12) of the coherent superposition state, which is related to the superposition coefficients *s*_*j*_ and the renormalized couplings *η*_*j*_ through Eq. (10). When the parameters of any zero-points *M*_*i*_ in Fig. 1 are adopted, Eq. (12) will be reduced to a relatively simple quantum state. We then select appropriate initial conditions to fix the coefficients *s*_*j*_, this simple quantum state can become a SCDT state or one of QDSLSs. The QDSLSs contain the CDT single states and NOON states. Because any SCDT state describes the Rabi oscillation between two or three QDSLSs and any one of the QDSLSs is associated with a set of fixed system parameters, we can adjust the driving parameters to prepare the QDSLSs and to control the transitions between QDSLSs transparently, through some interim SCDT states.

### Preparation of QDSLSs

We at first seek the CDT single states and NOON state with the same Floquet quasienergy by setting the parameters associated with the different zero-points of renormalized couplings *η*_*j*_ and the different superposition coefficients *s*_*j*_. These QDSLSs are derived for the following three cases:

*Case 1*: *η*_1_ = *η*_2_ = 0 of the zero-points *M*_1_, *M*_2_ and *M*_4_. In such a case, the superposition state (12) of Floquet states is reduced to a simple form [see Eq. (A1) in the Appendix]. We then select the initial conditions *P*_0_(0) = 1, *P*_*i*≠0_(0) = 0 or *P*_1_(0) = 1, *P*_*i*≠1_(0) = 0 to fix *s*_3_ = *s*_4_ = 0 and ${s}_{1}=\frac{1}{\sqrt{2}}={s}_{2}$ or −*s*_2_. Substituting them into Eq. (A1), respectively, results in the two *CDT single states*

13 $$|{\psi }_{03}(t)\rangle ={e}^{-3i[(n+l)\omega t+\frac{{\varepsilon }_{1}}{\omega }\sin (\omega t)]}|0,3\rangle ,$$

14 $$|{\psi }_{12}(t)\rangle ={e}^{-i[(n+l)\omega t+\frac{{\varepsilon }_{1}}{\omega }\sin (\omega t)]}|1,2\rangle .$$

They are different superpositions of the Floquet states *φ*_1_ and *φ*_2_ in Eqs (11) and (12) with the same quasienergy *E*_1_ = *E*_2_ = 0 and the amplitudes ${A}_{\mathrm{1,2}}={B}_{1}=-\,{B}_{2}=\frac{1}{\sqrt{2}}$, *C*_1,2_ = *D*_1,2_ = 0.

*Case 2*: *η*_2_ = *η*_3_ = 0 of the zero-point *M*_7_. Similarly, the reduced superposition state is given by Eq. (A2) in the Appendix. Applying the initial conditions *P*_2_(0) = 1, *P*_*i*≠2_(0) = 0 or *P*_3_(0) = 1, *P*_*i*≠3_(0) = 0 to fix *s*_3_ = *s*_4_ = 0 and ${s}_{2}=\frac{1}{\sqrt{2}}=\pm {s}_{1}$, respectively, Eq. (A2) becomes the other *CDT single states* with zero quasienergy

15 $$|{\psi }_{21}(t)\rangle ={e}^{-i[(n-l)\omega t-\frac{{\varepsilon }_{1}}{\omega }\sin (\omega t)]}|2,1\rangle ,$$

16 $$|{\psi }_{30}(t)\rangle ={e}^{-3i[(n-l)\omega t-\frac{{\varepsilon }_{1}}{\omega }\sin (\omega t)]}|3,0\rangle .$$

*Case 3*: *η*_1_ = *η*_3_ = 0 of the zero-point *M*_8_. In this case, the reduced superposition state is given by Eq. (A3) in the Appendix. For the initial constants *s*_3_ = *s*_4_ = 0 and *s*_1_ ≠ ±*s*_2_, Eq. (A3) becomes the general *NOON state*

17 $$\begin{matrix}\left|{\psi }_{NOON}(t)\right\rangle  & = & \frac{1}{\sqrt{2}}({s}_{1}+{s}_{2}){e}^{-3i[(n+l)\omega t+\frac{{\varepsilon }_{1}}{\omega }\sin (\omega t)]}\left|0,3\right\rangle \\  &  & +\frac{1}{\sqrt{2}}({s}_{1}-{s}_{2}){e}^{-3i[(n-l)\omega t-\frac{{\varepsilon }_{1}}{\omega }\sin (\omega t)]}\left|3,0\right\rangle \end{matrix}$$

with zero quasienergy and the invariant populations ${P}_{0}(t)={P}_{0}\mathrm{(0)}=\frac{1}{2}{|{s}_{1}+{s}_{2}|}^{2},{P}_{3}(t)={P}_{3}\mathrm{(0)}=\frac{1}{2}{|{s}_{1}-{s}_{2}|}^{2}$. The normalization implies the relation $\frac{1}{2}{|{s}_{1}+{s}_{2}|}^{2}+\frac{1}{2}{|{s}_{1}-{s}_{2}|}^{2}=1$ between *s*_1_ and *s*_2_. When *s*_1_ = 1, *s*_2_ = 0 and *s*_1_ = 0, *s*_2_ = 1 are selected respectively, Eq. (17) gives two maximal entangled states^23^ of the two modes^19,21^.

The five SLSs of Eqs (13–17) are different superpositions of the Floquet states *φ*_1_ and *φ*_2_ with the same quasienergy *E*_1_ = *E*_2_ = 0, so they are called the QDSLSs.

### Preparation of SCDT states

It is well known that any one of the above-mentioned QDSLSs corresponds to a special atomic distribution. Therefore, the Rabi oscillation between the QDSLSs means the periodic population transfer. The SCDT states which describe such Rabi oscillations can serve as the interim states to realize the quantum transitions between the QDSLSs. In this subsection, six SCDT states are derived from Eq. (12) for the following five cases.

*Case 1*: *η*_1_ = 0 of the zero-point *M*_5_. In this case, Eq. (10) gives the quasienergies *E*_1,2_ = 0, ${E}_{\mathrm{3,4}}=\pm \frac{1}{2}\sqrt{4{\eta }_{2}^{2}+3{\eta }_{3}^{2}}$ and the corresponding constants *A*_*j*_, *B*_*j*_, *C*_*j*_, *D*_*j*_ for *j* = 1, 2, 3, 4. Consequently, Eq. (12) becomes the SCDT state^49^,

18 $$\begin{matrix}\left|{\psi }_{122130}(t)\right\rangle  & = & [\frac{\sqrt{3}{\eta }_{3}}{\sqrt{4{\eta }_{2}^{2}+3{\eta }_{3}^{2}}}({s}_{2}-{s}_{1})+\frac{\sqrt{2}{\eta }_{2}}{\sqrt{4{\eta }_{2}^{2}+3{\eta }_{3}^{2}}}({s}_{3}{e}^{-i{E}_{3}t}-{s}_{4}{e}^{i{E}_{3}t})]\\  &  & \times {e}^{-i[(n+l)\omega t+\frac{{\varepsilon }_{1}}{\omega }\sin (\omega t)]}\left|1,2\right\rangle \\  &  & +[\frac{2{\eta }_{2}}{\sqrt{4{\eta }_{2}^{2}+3{\eta }_{3}^{2}}}({s}_{1}-{s}_{2})+\frac{\sqrt{3}{\eta }_{3}}{\sqrt{8{\eta }_{2}^{2}+6{\eta }_{3}^{2}}}({s}_{3}{e}^{-i{E}_{3}t}-{s}_{4}{e}^{i{E}_{3}t})]\\  &  & \times {e}^{-3i[(n-l)\omega t-\frac{{\varepsilon }_{1}}{\omega }\sin (\omega t)]}\left|3,0\right\rangle \\  &  & -\frac{1}{\sqrt{2}}({s}_{3}{e}^{-i{E}_{3}t}+{s}_{4}{e}^{i{E}_{3}t}){e}^{-i[(n-l)\omega t-\frac{{\varepsilon }_{1}}{\omega }\sin (\omega t)]}\left|2,1\right\rangle .\end{matrix}$$

It describes the Rabi oscillation among the Fock states |1, 2〉, |2, 1〉 and |3, 0〉, and means the corresponding population transfer. Because Eq. (18) does not contain the Fock state |0, 3〉, so it implies the SCDT from any one of the Fock states |1, 2〉, |2, 1〉, |3, 0〉 to the |0, 3〉 state.

*Case 2*: *η*_2_ = 0 of the zero-point *M*_3_. The reduced superposition state of Floquet states is given by Eq. (A4) in the Appendix. For *s*_3_ = *s*_4_ = 0 and *s*_1_, *s*_2_ ≠ 0, Eq. (A4) becomes the SCDT state

19 $$\begin{matrix}\left|{\psi }_{0312}(t)\right\rangle  & = & \frac{1}{\sqrt{2}}({s}_{1}{e}^{-i{E}_{1}t}+{s}_{2}{e}^{i{E}_{1}t}){e}^{-3i[(n+l)\omega t+\frac{{\varepsilon }_{1}}{\omega }\sin (\omega t)]}\left|0,3\right\rangle \\  &  & +\frac{1}{\sqrt{2}}({s}_{1}{e}^{-i{E}_{1}t}+{s}_{2}{e}^{i{E}_{1}t}){e}^{-i[(n+l)\omega t+\frac{{\varepsilon }_{1}}{\omega }\sin (\omega t)]}\left|1,2\right\rangle ,\end{matrix}$$

which describes the Rabi oscillation between the Fock states |0, 3〉 and |1, 2〉, and infers for the SCDT from any one of the states |0, 3〉 and |1, 2〉 to the states |3, 0〉 and |2, 1〉.

When *s*_1_ = *s*_2_ = 0 and *s*_3_, *s*_4_ ≠ 0 are set, Eq. (A4) of the Appendix becomes the SCDT state

20 $$\begin{matrix}\left|{\psi }_{2130}(t)\right\rangle  & = & \frac{1}{\sqrt{2}}({s}_{3}{e}^{-i{E}_{3}t}+{s}_{4}{e}^{i{E}_{3}t}){e}^{-i[(n-l)\omega t-\frac{{\varepsilon }_{1}}{\omega }\sin (\omega t)]}\left|2,1\right\rangle \\  &  & +\frac{1}{\sqrt{2}}(-\,{s}_{3}{e}^{-i{E}_{3}t}+{s}_{4}{e}^{i{E}_{3}t}){e}^{-3i[(n-l)\omega t-\frac{{\varepsilon }_{1}}{\omega }\sin (\omega t)]}\left|3,0\right\rangle .\end{matrix}$$

This state means the Rabi oscillation between states |2, 1〉 and |3, 0〉, and the SCDT from any one of states |2, 1〉 and |3, 0〉 to states |0, 3〉 and |1, 2〉.

*Case 3*: *η*_3_ = 0 of the zero-point *M*_6_. This case means *E*_1,2_ = 0, ${E}_{3,4}=\pm \frac{1}{2}\sqrt{3{\eta }_{1}^{2}+4{\eta }_{2}^{2}}$, so Eq. (12) becomes the SCDT state

21 $$\begin{matrix}\left|{\psi }_{031221}(t)\right\rangle  & = & [\frac{2{\eta }_{2}}{\sqrt{3{\eta }_{1}^{2}+4{\eta }_{2}^{2}}}({s}_{1}+{s}_{2})+\frac{\sqrt{3}{\eta }_{1}}{\sqrt{6{\eta }_{1}^{2}+8{\eta }_{2}^{2}}}({s}_{3}{e}^{-i{E}_{3}t}+{s}_{4}{e}^{i{E}_{3}t})]\\  &  & \times {e}^{-3i[(n+l)\omega t+\frac{{\varepsilon }_{1}}{\omega }\sin (\omega t)]}\left|0,3\right\rangle \\  &  & +[-\,\frac{\sqrt{3}{\eta }_{1}}{\sqrt{3{\eta }_{1}^{2}+4{\eta }_{2}^{2}}}({s}_{1}+{s}_{2})+\frac{\sqrt{2}{\eta }_{2}}{\sqrt{3{\eta }_{1}^{2}+4{\eta }_{2}^{2}}}({s}_{3}{e}^{-i{E}_{3}t}+{s}_{4}{e}^{i{E}_{3}t})]\\  &  & \times {e}^{-i[(n-l)\omega t-\frac{{\varepsilon }_{1}}{\omega }\sin (\omega t)]}\left|2,1\right\rangle \\  &  & -\frac{1}{\sqrt{2}}({s}_{3}{e}^{-i{E}_{3}t}-{s}_{4}{e}^{i{E}_{3}t}){e}^{-i[(n+l)\omega t+\frac{{\varepsilon }_{1}}{\omega }\sin (\omega t)]}\left|1,2\right\rangle .\end{matrix}$$

It means the Rabi oscillation among states |0, 3〉, |1, 2〉 and |2, 1〉, and the SCDT from any one of states |0, 3〉, |1, 2〉 and |2, 1〉 to state |3, 0〉.

*Case 4*: *η*_1_ = *η*_3_ = 0 of the zero-point *M*_8_. Here the parameters are the same as those of the NOON state case. We apply the different coefficients *s*_1_ = *s*_2_ = 0 and *s*_3_, *s*_4_ ≠ 0 to Eq. (A3) of the Appendix, the latter becomes the SCDT state

22 $$\begin{matrix}\left|{\psi }_{1221}(t)\right\rangle  & = & \frac{1}{\sqrt{2}}({s}_{3}{e}^{-i{E}_{3}t}-{s}_{4}{e}^{i{E}_{3}t}){e}^{-i[(n+l)\omega t+\frac{{\varepsilon }_{1}}{\omega }\sin (\omega t)]}\left|1,2\right\rangle \\  &  & -\frac{1}{\sqrt{2}}({s}_{3}{e}^{-i{E}_{3}t}+{s}_{4}{e}^{i{E}_{3}t}){e}^{-i[(n-l)\omega t-\frac{{\varepsilon }_{1}}{\omega }\sin (\omega t)]}\left|2,1\right\rangle ,\end{matrix}$$

which describes the Rabi oscillation between states |1, 2〉 and |2, 1〉, and the SCDT from any one of states |1, 2〉 and |2, 1〉 to states |0, 3〉 and |3, 0〉.

*Case 5*: *η*_1_ = *η*_3_ ≈ 0.302 and *η*_2_ ≈ 1.996 for *m* = 2, *n* = 1, *l* = 0, Ω_0_ = 1, *ω* = 20, *ε*_1_ = 0.05*ω* and Ω_1_ = 0.3. In order to seek a SCDT state describing the Rabi oscillation between states |0, 3〉 and |3, 0〉, we require nonzero coupling constants to make ${b}_{1}^{^{\prime} }(t)\approx {b}_{2}^{^{\prime} }(t)\approx 0$ in Eq. (12) for any time. Such special couplings are found numerically as *η*_1_ = *η*_3_ ≈ 0.302 and *η*_2_ ≈ 1.996 (see Sec. B of the Appendix). In this case, if the initial state is taken as |0, 3〉, Eq. (12) is approximately the SCDT state

23 $$\begin{matrix}\left|{\psi }_{0330}(t)\right\rangle  & \approx  & \sum _{j=1}^{4}{s}_{j}{A}_{j}{e}^{-i{E}_{j}t}{e}^{-3i[(n+l)\omega t+\frac{{\varepsilon }_{1}}{\omega }\sin (\omega t)]}\left|0,3\right\rangle \\  &  & +\sum _{j=1}^{4}{s}_{j}{D}_{j}{e}^{-i{E}_{j}t}{e}^{-3i[(n-l)\omega t-\frac{{\varepsilon }_{1}}{\omega }\sin (\omega t)]}\left|3,0\right\rangle \end{matrix}$$

for *s*_1_ = *s*_2_ ≈ −0.701, *s*_3_ = *s*_4_ ≈ 0.09, *A*_1,2_ = *D*_1_ = −*D*_2_ ≈ −0.701, *A*_3,4_ = −*D*_3_ = *D*_4_ ≈ 0.09, *E*_1_ = −*E*_2_ ≈ 0.034 and *E*_3_ = −*E*_4_ ≈ 2.029. This state means that the Rabi oscillation between states |0, 3〉 and |3, 0〉 is allowable and the SCDT from any one of states |0, 3〉 and |3, 0〉 to any one of states |1, 2〉 and |2, 1〉 can occur.

### Quasienergy spectra analysis

**Figure 2:**
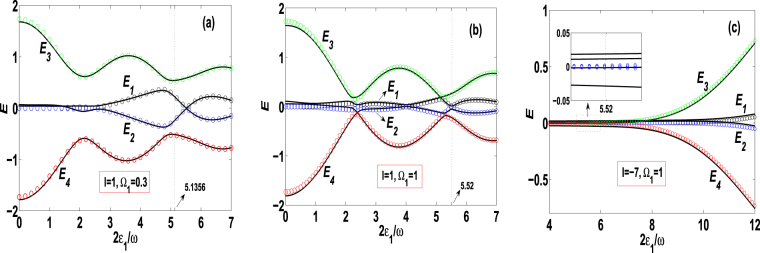
Quasienergies as functions of the driving parameter, *E*_*j*_ = *E*_*j*_(2*ε*_1_/*ω*) for *ω* = 20, Ω_0_ = 1, *m* = *n* = 1 and (**a**) *l* = 1, Ω_1_ = 0.3; (**b**) *l* = 1, Ω_1_ = 1; (**c**) *l* = −7, Ω_1_ = 1. Hereafter, circles label the analytical results from Eq. (10) and solid curves denote the numerical correspondences, unless it is specially indicated.

We have analytically obtained the Floquet quasienergies in Eq. (10), which directly affect the Floquet states (11) and their superposition state (12). To see the effect of the quasienergies on the quantum states, as functions of the driving parameters the Floquet quasienergies *E*_*j*_ = *E*_*j*_(2*ε*_1_/*ω*) for *j* = 1, 2, 3, 4 are plotted by the circles in Fig. 2, where the parameters are taken as *ω* = 20, Ω_0_ = 1, *m* = *n* = 1 (*ω*′ = *U* = *ω*), and (a) *l* = 1 (*ε*_0_ = *ω*), Ω_1_ = 0.3; (b) *l* = 1 (*ε*_0_ = *ω*), Ω_1_ = 1; and (c) *l* = −7 (*ε*_0_ = −7*ω*), Ω_1_ = 1. Due to the Hamiltonian (2) is time periodic, we can introduce the Hermitian operator^5,36^ ${\mathcal H} (t)=\hat{H}(t)-i\frac{\partial }{\partial t}$ and numerically solve the eigenvalue equation ${\mathcal H} (t)|{\varphi }_{j}(t)\rangle =E|{\varphi }_{j}(t)\rangle$ of the Floquet state |*φ*_*j*_(*t*)〉. The quasienergies *E* = *E*_*j*_(2*ε*_1_/*ω*) for *j* = 1, …, 4 can be easily obtained from the eigenvalue equation for the analytically used parameters. The numerical results are shown by the solid curves of Fig. 2. Clearly, both the analytical and numerical results are in good agreement.

In Fig. 2(a), the quasienergies $$\begin{matrix}{E}_{\mathrm{1,2}}(0)=0, & {E}_{3,4}(0)=\pm \sqrt{3}\end{matrix}$$ are associated with the parameters of the point *M*_1_(2*ε*_1_/*ω*, *η*_1_, *η*_2_, *η*_3_) = *M*_1_(0, 0, 0, 2) in Fig. 1(a) and the quasi-degenerate CDT single states (13) and (14). The quasienergies *E*_1,2_(5.1356) ≈ ±0.23, *E*_3,4_(5.1356) ≈ ±0.53 correspond to the zero-point *M*_3_(5.1356, 0.61, 0, −0.26) in Fig. 1(a) and the SCDT states (19) and (20). Therefore, we can control the quantum transition from one of the QDSLSs (13) and (14) to the state (19) by setting the suitable initial conditions and adjusting the driving parameter 2*ε*_1_/*ω* from 0 to 5.1356.

In Fig. 2(b) with *l* = 1, the quasienergies $$\begin{matrix}{E}_{\mathrm{1,2}}(0)=0, & {E}_{\mathrm{3,4}}(0)=\pm \sqrt{3}\end{matrix}$$ are associated with the parameters of the point *M*_4_(0, 0, 0, 2) in Fig. 1(b) and the states (13) and (14). However, the quasienergies *E*_1,2_(5.52) = 0, *E*_3,4_(5.52) ≈ ±0.25 correspond to the point *M*_6_(5.52, 0.22, −0.16,0) in Fig. 1(b) and the different state (21). Therefore, the adjustment of 2*ε*_1_/*ω* from 0 to 5.52 will cause the transfer from state (13) or (14) to state (21).

In Fig. 2(c) with *l* = −7, the quasienergies *E*_1,2_(5.52) = 0, *E*_3,4_(5.52) = ±0.02 are associated with the parameters of the point *M*_7_(5.52, 0.001, 0, 0) in Fig. 1(c) and the states of Eqs (15) and (16). Thus, from Fig. 2(b) and (c) we find that when the value of *l* is adjusted from 1 to −7, the corresponding quantum transition occurs from Eqs (21) to (15) for the same value 2*ε*_1_/*ω* = 5.52. In the inset, we show that the absolute value of maximum deviation between the analytical results (circles) and the numerical ones (solid curves) is less than 0.05. Such a deviation can be decreased by analytically taking into account the second-order effect of quantum tunneling^50^.

The detailed control proposals of the quantum transitions between the QDSLSs will be given in next subsection.

### Manipulating transitions between QDSLSs without quasi-level difference

**Figure 3:**
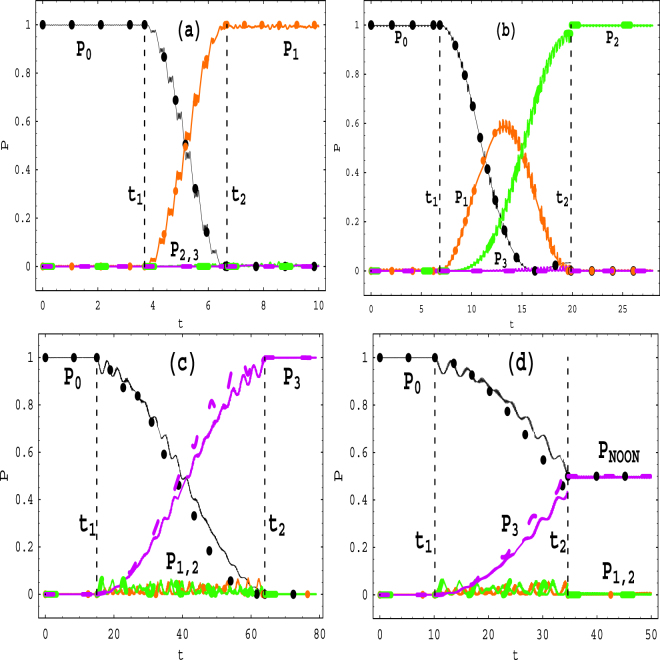
Time evolutions of the transition probabilities from the initial SLS |*ψ*_03_(*t*)〉 to other final SLSs for the parameters *ω* = 20, Ω_0_ = 1, *n* = 1, and (**a**) Ω_1_ = 0.3, *m* = *l* = 1, *ε*_1_ = 0 (in the time intervals $0\le t< {t}_{1}=\frac{47\pi }{40}$ and $t\ge {t}_{2}=\frac{17\pi }{8}$) and *ε*_1_ = 2.5678*ω* (in interval ${t}_{1}=\frac{47\pi }{40}\le t< {t}_{2}=\frac{17\pi }{8}$); (**b**) Ω_1_ = 1, *m* = 1, (*l*, *ε*_1_) = (1, 0) (in $0\le t< {t}_{1}=\frac{87\pi }{40}$), (*l*, *ε*_1_) = (1, 2.76*ω*) (in ${t}_{1}=\frac{87\pi }{40}\le t< {t}_{2}=\frac{253\pi }{40}$), (*l*, *ε*_1_) = (−7, 2.76*ω*) (in $t\ge {t}_{2}=\frac{253\pi }{40}$); (**c**) Ω_1_ = 0.3, *m* = 2, *ε*_1_ = 0.05*ω*, and *l* = 2 (in $0\le t< {t}_{1}=\frac{191\pi }{40}$), *l* = 0 (in ${t}_{1}=\frac{191\pi }{40}\le t< {t}_{2}=\frac{163\pi }{8}$), *l* = −2 (in $t\ge {t}_{2}=\frac{163\pi }{8}$); (**d**) Ω_1_ = 0.3, *m* = 2, (*l*, *ε*_1_) = (2, 0.05*ω*) (in $0\le t< {t}_{1}=\frac{129\pi }{40}$), (*l*, *ε*_1_) = (0, 0.05*ω*) (in ${t}_{1}=\frac{129\pi }{40}\le t< {t}_{2}=\frac{441\pi }{40}$), (*l*, *ε*_1_) = (0, 2.64*ω*) (in $t\ge {t}_{2}=\frac{441\pi }{40}$). Hereafter, the big circular points (online black), small circular points (online orange), thick dashed line (online green) and thin dashed line (online purple) label the analytical results of probabilities *P*_0_, *P*_1_, *P*_2_, *P*_3_, respectively, and the solid curves denote the numerical correspondences.

**Figure 4:**
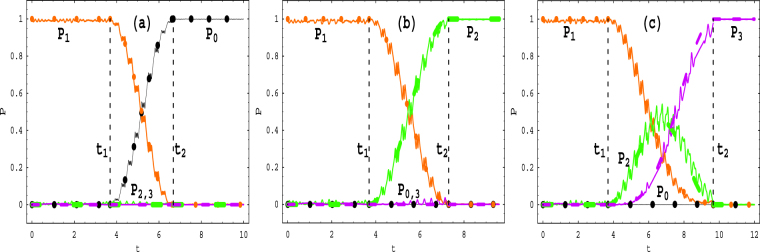
Time evolutions of the transition probabilities from the initial SLS |*ψ*_12_(*t*)〉 to other final SLSs. (**a**) The parameters are the same as those of Fig. 3(a); (**b**) The parameters are the same as those of Fig. 3(a) except for Ω_1_ = 0.6, and (*l*, *ε*_1_) obeying (*l*, *ε*_1_) = (1, 1.2025*ω*) in ${t}_{1}=\frac{47\pi }{40}\le t< {t}_{2}=\frac{93\pi }{40}$, (*l*, *ε*_1_) = (−4, 1.2025*ω*) in $t\ge {t}_{2}=\frac{93\pi }{40}$; (**c**) The parameters are the same as those of Fig. 3(a) except for Ω_1_ = 0.5, and (*l*, *ε*_1_) obeying (*l*, *ε*_1_) = (1, *ω*) in ${t}_{1}=\frac{47\pi }{40}\le t< {t}_{2}=\frac{123\pi }{40}$, (*l*, *ε*_1_) = (−3, *ω*) in $t\ge {t}_{2}=\frac{123\pi }{40}$.

**Figure 5:**
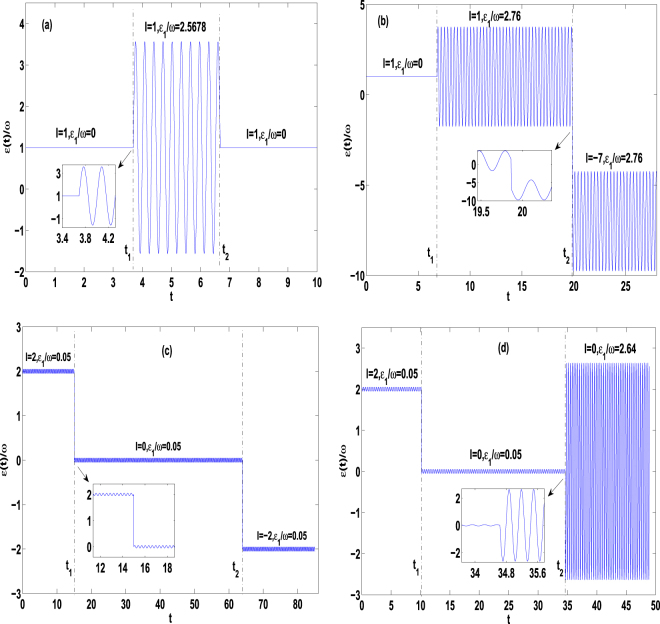
Temporal sequences of the time-dependent bias *ε*(*t*) with the parameters of Fig. 5(a,b,c,d) being the same as those of Fig. 3(a,b,c,d), respectively, in the corresponding time intervals. The insets indicate the evolution details around several operation times. In (**a**), the driving of amplitude *ε*_1_ = 2.5678*ω* is continuously added to the constant bias *ε*_0_ = 1*ω*. In (**b**,**c** and **d**), the static bias is adjusted between the discrete values^6^, which leads to discontinuous changes of *ε*(*t*).

Given the above-mentioned five QDSLSs and six SCDT states, and according to the quasienergy spectra analysis, we can transparently perform manipulations of the transitions between QDSLSs. Generally, the control proposal is designed as the following: Firstly, we fix the parameters (*ω*, *n*, *m*, *l*, Ω_*i*_, *ε*_*i*_) to prepare an initial SLS 1, then selecting a final SLS 2 and the interim SCDT state 3 which oscillates between SLS 1 and SLS 2. Secondly, we change the ac field strength *ε*_1_ or dc field strength *l*(*ω*) at an appropriate time *t*_1_ to make the state transition from the SLS 1 to the state 3; Finally, when the oscillating state 3 reaches the SLS 2, we perform the adjustment of *ε*_1_ or *l* again to create the transition from state 3 to SLS 2. In Table 1 and Figs 3 and 4, we show seven control proposals of quantum transitions from the initial SLS |*ψ*_03_(*t*)〉 (or |*ψ*_12_(*t*)〉) to any different final SLS, where *P*_*i*_ denotes the probability of *i* particle(s) being in the left well and (3 − *i*) particle(s) occupying the right well. In Fig. 5 we illustrate that in the four control proposals of Fig. 3 how the time-dependent bias of Eq. (4) can be adjusted experimentally.

**Table 1:** Several control proposals of quantum transitions between QDSLSs for some initially fixed parameter sets (*n*, *m*, Ω_*i*_).

Initial SLSs $(0\sim {t}_{1},l,\frac{{\varepsilon }_{1}}{\omega })$	SCDT states $(\sim {t}_{2},l,\frac{{\varepsilon }_{1}}{\omega })$	Final SLSs $(\ge {t}_{2},l,\frac{{\varepsilon }_{1}}{\omega })$	Figures
$|{\psi }_{03}(t)\rangle (0\sim \frac{47\pi }{40},1,0)$	$|{\psi }_{0330}(t)\rangle (\sim \frac{17\pi }{8},1,2.5678)$	$|{\psi }_{12}(t)\rangle (\ge \frac{17\pi }{8},1,0)$	Fig. 3(a)
$|{\psi }_{03}(t)\rangle (0\sim \frac{87\pi }{40},1,0)$	$|{\psi }_{031221}(t)\rangle (\sim \frac{253\pi }{40},1,2.76)$	$|{\psi }_{21}(t)\rangle (\ge \frac{253\pi }{40},-\,7,2.76)$	Fig. 3(b)
$|{\psi }_{03}(t)\rangle (0\sim \frac{191\pi }{40},2,0.05)$	$|{\psi }_{0330}(t)\rangle (\sim \frac{163\pi }{8},0,0.05)$	$|{\psi }_{30}(t)\rangle (\ge \frac{163\pi }{8},-\,2,0.05)$	Fig. 3(c)
$|{\psi }_{03}(t)\rangle (0\sim \frac{129\pi }{40},2,0.05)$	$|{\psi }_{0330}(t)\rangle (\sim \frac{441\pi }{40},0,0.05)$	$|{\psi }_{NOON}(t)\rangle (\ge \frac{441\pi }{40},0,2.64)$	Fig. 3(d)
$|{\psi }_{12}(t)\rangle (0\sim \frac{47\pi }{40},1,0)$	$|{\psi }_{0312}(t)\rangle (\sim \frac{17\pi }{8},1,2.5678)$	$|{\psi }_{03}(t)\rangle (\ge \frac{17\pi }{8},1,0)$	Fig. 4(a)
$|{\psi }_{12}(t)\rangle (0\sim \frac{47\pi }{40},1,0)$	$|{\psi }_{1221}(t)\rangle (\sim \frac{93\pi }{40},1,1.2025)$	$|{\psi }_{21}(t)\rangle (\ge \frac{93\pi }{40},-\,4,1.2025)$	Fig. 4(b)
$|{\psi }_{12}(t)\rangle (0\sim \frac{47\pi }{40},1,0)$	$|{\psi }_{122130}(t)\rangle (\sim \frac{123\pi }{40},1,1)$	$|{\psi }_{30}(t)\rangle (\ge \frac{123\pi }{40},-\,3,1)$	Fig. 4(c)

As an example, we first consider the proposal of the second line in Table 1, namely the transition from |*ψ*_03_(*t*)〉 of Eq. (13) to |*ψ*_12_(*t*)〉 of Eq. (14). The corresponding time evolutions of the probabilities are plotted in Fig. 3(a), where we initially place the three bosons in right well with *P*_0_(0) = 1 and *P*_*i*≠*o*_(0) = 0. By taking the parameters *ω* = 20, *m* = *n* = *l* = 1 (*ω*′ = *U* = *ε*_0_ = *ω*), Ω_0_ = 1, Ω_1_ = 0.3, *ε*_1_ = 0 to yield the renormalized couplings *η*_1_ = *η*_2_ = 0 of the zero-point *M*_1_ in the inset of Fig. 1(a), we prepare the initial SLS 1 as |*ψ*_03_(*t*)〉. Then at a selected time ${t}_{1}=\frac{47\pi }{40}$ which obeys the continuity condition *ε*(*t*_1_) = *ε*_0_ in Eq. (4), we change the driving strength *ε*_1_ from 0 to 2.5678*ω* to fit *η*_1_ ≠ 0, *η*_2_ = 0 of the zero-point *M*_3_ in Fig. 1(a) and to make the transition from |*ψ*_03_(*t*)〉 to the SCDT state 3 |*ψ*_0312_(*t*)〉 of Eq. (19). In the latter state, the system performs the Rabi oscillation between |0, 3〉 and |1, 2〉, as shown in Fig. 3(a). When the time approaches ${t}_{2}=\frac{17\pi }{8}$ and the probability *P*_1_(*t*) of state |1, 2〉 reaches 1, we return the driving strength to *ε*_1_ = 0 that creates the final SLS 2 |*ψ*_12_(*t*)〉 with one atom in the left well and two atoms in the right well. In such a transition, one atom tunnels from the right well to left well. *The analytical result (circular points) is conformed by the numerical one* (*solid curves*) *based on* Eq. (7)*, and perfect agreement is shown in* Fig. 3(a).

Similarly, the control proposals of the quantum transitions from the initial SLS |*ψ*_03_(*t*)〉 to different final SLSs |*ψ*_21_(*t*)〉, |*ψ*_30_(*t*)〉 and |*ψ*_*NOON*_(*t*)〉 are also given in Fig. 3(b,c and d), respectively. In Fig. 4, we give the other control proposals of the quantum transitions from the initial SLS |*ψ*_12_(*t*)〉 to one of the final SLSs |*ψ*_03_(*t*)〉, |*ψ*_21_(*t*)〉 and |*ψ*_30_(*t*)〉. Because the left-right symmetry of the system, the quantum transitions starting from the initial SLSs |*ψ*_30_(*t*)〉 and |*ψ*_21_(*t*)〉 are equivalent to the above considered transitions. Therefore, it is asserted that we can achieve the quantum transitions between arbitrary two SLSs analytically and numerically. Particularly, the outcome of the predicted transitions presented in our paper is insensitive to slight deviations of the parameters, which means that the manipulating scheme for quantum transitions between QDSLSs are workable experimentally.

The above-mentioned control proposals can be performed experimentally. For instance, the time-dependent bias associated with Fig. 3(a) is shown schematically in Fig. 5(a), where the dc part *ε*_0_ is kept as *ε*_0_ = *lω* for *l* = 1 and *t* ≥ 0, and the ac field with amplitude *ε*_1_ = 2.5678*ω* for *ω* = 20 is applied in the time interval *t*_1_ ≤ *t* ≤ *t*_2_. *The driving amplitude ε*_1_ *can be adjusted in an experiment* by periodically varying the magnetic field gradient applied along the *x* direction^7,8^ or by employing the mirrors mounted on piezoelectric actuators that allow one to sinusoidally shake each lattice back and forth^9^. The continuity of *ε*(*t*) at the operation times *t*_*i*_ for *i* = 1, 2 leads to the smooth modulations.

The corresponding temporal sequences of the bias associated with Fig. 3(b,c and d) are shown schematically in Fig. 5(b,c and d), respectively, where the static bias *ε*_0_ is changed between two discrete values for fitting the resonance condition *ε*_0_ = *lω*. *Such nonadiabatic adjustments between discontinuous values of the static bias are experimentally feasible, since the similar experimental operations have been implemented* ^6^.

## Discussion

We have investigated coherent control of the quantum transitions between *quasi-degenerate stationary-like states* (QDSLSs) without detectable absorption or emission, which contain the quasi-degenerate CDT single states and NOON state by using three bosons held in a depth-tilt combined-modulated double-well potential. Within the high-frequency approximation and for the multiple-resonance conditions, we have analytically obtained the Floquet solutions in Eq. (11) and quasienergies in Eq. (10). Employing the Floquet states as a set of complete bases, we construct general coherent superposition state (12) of the Floquet states. Given the analytical solutions, we demonstrate that combining some special values of the driving parameters with the appropriate initial conditions can lead to five different QDSLSs and six SCDT states. The latter states describe the Rabi oscillations between two or three QDSLSs. Applying them as the interim states, we give seven control proposals of quantum transitions between QDSLSs in Table 1. The quasienergy spectra analysis based on Fig. 2 supports the existence of the QDSLSs and indicates the feasibility of quantum transitions between the QDSLSs. The analytical results are verified by the numerical calculations based on the exact Eq. (7) and perfect agreements are shown in Figs 2, 3 and 4.

According to our control schemes, we can achieve the quantum transitions between any pair of QDSLSs analytically and numerically. The transitions between QDSLSs without quasi-level difference are equivalent to the related population transfers, and can thereby be observed and controlled by adjusting the atomic distributions of the initial and final states in the current experimental setup^6^. We also expect possible applications of the scheme to the preparation of robust quantum entangled states and to the quantum information processing. In fact, a double-well trapped many-boson system can be divided into two subsystems *A* and *B* and the corresponding bipartite entangled states have been investigated^19–21^, which can be used to encode the qubit^22^. Noticing the indistinguishability of identical bosons, we can divide the considered three-boson system into the subsystem *A* of a single-boson and subsystem *B* of a pair-boson. Then by employing the correspondence between the double wells 1, 2 and the two internal states^25^ |−〉 and |+〉, the basis in Eq. (6) can be rewritten as |0, 3〉 = |+〉_*A*_|+〉_*B*_, |1, 2〉 = |−〉_*A*_|+〉_*B*_, |2, 1〉 = |+〉_*A*_|−〉_*B*_, |3, 0〉 = |−〉_*A*_|−〉_*B*_. The new expression of the basis is just the standard basis for a two-qubit system^51,52^. At *t* = 0 the NOON states of Eq. (17) with *s*_1_ = 1, *s*_2_ = 0 or *s*_1_ = 0, *s*_2_ = 1 is just one of the usual Bell basis, ${\psi }_{NOON\pm }(0)=\frac{1}{\sqrt{2}}(|0,3\rangle \pm |3,0\rangle )=\frac{1}{\sqrt{2}}({|+\rangle }_{A}{|+\rangle }_{B}\pm {|-\rangle }_{A}{|-\rangle }_{B})$, and the transition from the initial CDT single state *ψ*_03_(*t*_*i*_) to the final NOON state *ψ*_*NOON*_(*t*_*f*_) results in generation of the maximal entanglement. Thus we can use the considered system to simulate a two-qubit system and to seek the connections between the operations in Table 1 and the two-qubit logical gates^25,52^, and to investigate possible manipulations of the qubits aimed at quantum computing purposes and quantum information transfer between two qubits^25^. While the quasi-degeneracy of the QDSLSs enable us to suppress the decoherence from the spontaneous transitions.

## Methods

### Theoretical analysis

We first derive the Bose-Hubbard Hamiltonian (2) from the potential (1) by using the tight-binding approximation. Then we employ the high-frequency limit to simplify the time-evolution equation (7) of the probability amplitudes as Eq. (8), leading to the Floquet states and quasienergies. Further application of the multiple-resonance conditions results in different QDSLSs and SCDT states for the different renormalized coupling constants. By adjusting the dc field strength *ε*_0_ or ac field strength *ε*_1_ of the time-dependent bias *ε*(*t*), we achieve the coherent manipulation to the quantum transition between arbitrary QDSLSs via an interim SCDT. The corresponding experimental proposals are suggested, as illustrated in Fig. 5.

### Numerical calculation

In addition to theoretical analysis, we have also carried out numerical computations to confirm the analytical results. By applying the MATLAB code to the eigenvalue equation $(\hat{H}(t)-i\frac{\partial }{\partial t})|{\varphi }_{j}(t)\rangle =E|{\varphi }_{j}(t)\rangle$ of the Floquet state |*φ*_*j*_(*t*)〉, we get the numerical results of Floquet quasienergies, as indicated by the solid curves of Fig. 2. Then we apply the MATHEMATICA code to the coupled equations (7), yielding the numerical results of the transition probabilities *P*_*i*_ shown by the solid curves of Figs 3 and 4. The numerical results and analytical ones are in perfect agreement.

## Electronic supplementary material

Supplementary Information
