# Supplementary material for: Coherent control of quasi-degenerate stationary-like states via multiple resonances
Source: Sci Rep. 2017 Feb 2;7:21. doi: 10.1038/s41598-017-00041-x (PMC5428370; doi:10.1038/s41598-017-00041-x)
Supplement: Supplementary file 1 — Supplementary Information [file 41598_2017_41_MOESM1_ESM.pdf]

Coherent control of quasi-degenerate stationary-like s-tates via multiple resonances

Yunrong Luo[1,2], Kuo Hai[1,2], Mingliang Zou[1] & Wenhua Hai[1,2,⋆]

APPENDIX. Derivations of QDSLSs and SCDT states

A. Derivations of QDSLSs. *Case 1*: $\eta_1 = \eta_2 = 0$.

In such a case, from Eq. (10) we easily obtain $E_{1,2} = 0$, $E_{3,4} = \pm\frac{\sqrt{3}|\eta_3|}{2}$, $A_{1,2} = B_1 = C_{3,4} = D_4 = -B_2 = -D_3 = \frac{1}{\sqrt{2}}$, $A_{3,4} = B_{3,4} = C_{1,2} = D_{1,2} = 0$. Combining Eq. (11) with Eq. (12), the superposition state of Floquet states is reduced to

$$\begin{aligned}|\psi(t)\rangle \;=\;& \frac{1}{\sqrt{2}}(s_1+s_2)e^{-3i[(n+l)\omega t+\frac{\varepsilon_1}{\omega}\sin(\omega t)]}|0,3\rangle + \frac{1}{\sqrt{2}}(s_1-s_2)e^{-i[(n+l)\omega t+\frac{\varepsilon_1}{\omega}\sin(\omega t)]}|1,2\rangle \\ &+\frac{e^{iE_3t}}{\sqrt{2}}(s_3e^{-2iE_3t}+s_4)e^{-i[(n-l)\omega t-\frac{\varepsilon_1}{\omega}\sin(\omega t)]}|2,1\rangle \\ &+\frac{e^{iE_3t}}{\sqrt{2}}(-s_3e^{-2iE_3t}+s_4)e^{-3i[(n-l)\omega t-\frac{\varepsilon_1}{\omega}\sin(\omega t)]}|3,0\rangle. \qquad \text{(A1)}\end{aligned}$$

We select the initial conditions a) $P_0(0) = 1,\ P_{i\neq 0}(0) = 0$ or b) $P_1(0) = 1,\ P_{i\neq 1}(0) = 0$, which lead to $s_3 = s_4 = 0$ and a) $s_1 = \frac{1}{\sqrt{2}} = s_2$ or b) $s_1 = \frac{1}{\sqrt{2}} = -s_2$. Substituting them into Eq. (A1), results in the two *quasi-degenerate CDT single states* $|\psi_{03}(t)\rangle$ and $|\psi_{12}(t)\rangle$ in Eqs. (13) and (14), respectively.

Case 2: $\eta_2 = \eta_3 = 0$.

Similarly, when $\eta_2 = \eta_3 = 0$ is set, Eqs. (10), (11) and (12) give the quasienergies $E_{1,2} = 0$,

$E_{3,4} = \pm\frac{\sqrt{3}|\eta_1|}{2}$ and the state

$$
\begin{aligned}
|\psi(t)\rangle &= \frac{1}{\sqrt{2}}(s_2+s_1)e^{-i[(n-l)\omega t-\frac{\varepsilon_1}{\omega}\sin(\omega t)]}|2,1\rangle + \frac{1}{\sqrt{2}}(s_2-s_1)e^{-3i[(n-l)\omega t-\frac{\varepsilon_1}{\omega}\sin(\omega t)]}|3,0\rangle \\
&+\frac{e^{iE_3 t}}{\sqrt{2}}(s_4+s_3 e^{-2iE_3 t})e^{-3i[(n+l)\omega t+\frac{\varepsilon_1}{\omega}\sin(\omega t)]}|0,3\rangle \\
&+\frac{e^{iE_3 t}}{\sqrt{2}}(s_4-s_3 e^{-2iE_3 t})e^{-i[(n+l)\omega t+\frac{\varepsilon_1}{\omega}\sin(\omega t)]}|1,2\rangle. \qquad \text{(A2)}
\end{aligned}
$$

Applying the initial conditions $P_2(0) = 1,\ P_{i\neq 2}(0) = 0$ or $P_3(0) = 1,\ P_{i\neq 3}(0) = 0$ to get $s_3 = s_4 = 0$ and $s_2 = \frac{1}{\sqrt{2}} = \pm s_1$, Eq. (A2) becomes the *quasi-degenerate CDT single states* $|\psi_{21}(t)\rangle$ and $|\psi_{30}(t)\rangle$ in Eqs. (15) and (16), respectively.

Case 3: $\eta_1 = \eta_3 = 0$.

Such a case means the quasienergies $E_{1,2} = 0$, $E_{3,4} = \pm|\eta_2|$, and leads the superposition state of Eq. (12) to the form

$$
\begin{aligned}
|\psi(t)\rangle &= \frac{1}{\sqrt{2}}(s_1+s_2)e^{-3i[(n+l)\omega t+\frac{\varepsilon_1}{\omega}\sin(\omega t)]}|0,3\rangle + \frac{1}{\sqrt{2}}(s_1-s_2)e^{-3i[(n-l)\omega t-\frac{\varepsilon_1}{\omega}\sin(\omega t)]}|3,0\rangle \\
&+\frac{e^{iE_3 t}}{\sqrt{2}}(s_3 e^{-2iE_3 t}-s_4)e^{-i[(n+l)\omega t+\frac{\varepsilon_1}{\omega}\sin(\omega t)]}|1,2\rangle \\
&-\frac{e^{iE_3 t}}{\sqrt{2}}(s_3 e^{-2iE_3 t}+s_4)e^{-i[(n-l)\omega t-\frac{\varepsilon_1}{\omega}\sin(\omega t)]}|2,1\rangle. \qquad \text{(A3)}
\end{aligned}
$$

When $s_3 = s_4 = 0$ and $s_1 \neq \pm s_2$ are taken, Eq. (A3) becomes the general *NOON-like state* $|\psi_{NOON}(t)\rangle$ with zero quasienergy in Eq. (17).

B. Derivations of SCDT states. *Case 1*: $\eta_1 = 0$.

In this case, Eq. (10) gives the quasienergies $E_{1,2} = 0$, $E_{3,4} = \pm\frac{1}{2}\sqrt{4\eta_2^2 + 3\eta_3^2}$ and the corresponding constants A_j, B_j, C_j, D_j for $j = 1, 2, 3, 4$. Consequently, Eq. (12) becomes the SCDT

state $|\psi_{122130}(t)\rangle$ in Eq. (18) which describes the Rabi oscillation among the Fock states $|1,2\rangle$, $|2,1\rangle$ and $|3,0\rangle$, and means the corresponding population transfer. Because Eq. (18) does not contain the Fock state $|0,3\rangle$, so it implies the SCDT from any one of the Fock states $|1,2\rangle,\ |2,1\rangle,\ |3,0\rangle$ to the $|0,3\rangle$ state.

Case 2: $\eta_2 = 0$.

Applying $\eta_2 = 0$ to Eqs. (10), (11) and (12) result in the quasienergies $E_{1,2} = \pm\frac{\sqrt{3\eta_1^2+3\eta_3^2-|3\eta_1^2-3\eta_3^2|}}{2\sqrt{2}}$, $E_{3,4} = \pm\frac{\sqrt{3\eta_1^2+3\eta_3^2+|3\eta_1^2-3\eta_3^2|}}{2\sqrt{2}}$ and the state

$$\begin{aligned}
|\psi(t)\rangle &= \frac{1}{\sqrt{2}}(s_1 e^{-iE_1 t} + s_2 e^{iE_1 t})e^{-3i[(n+l)\omega t+\frac{\varepsilon_1}{\omega}\sin(\omega t)]}|0,3\rangle \\
&+\frac{1}{\sqrt{2}}(-s_1 e^{-iE_1 t} + s_2 e^{iE_1 t})e^{-i[(n+l)\omega t+\frac{\varepsilon_1}{\omega}\sin(\omega t)]}|1,2\rangle \\
&+\frac{1}{\sqrt{2}}(s_3 e^{-iE_3 t} + s_4 e^{iE_3 t})e^{-i[(n-l)\omega t-\frac{\varepsilon_1}{\omega}\sin(\omega t)]}|2,1\rangle \\
&+\frac{1}{\sqrt{2}}(-s_3 e^{-iE_3 t} + s_4 e^{iE_3 t})e^{-3i[(n-l)\omega t-\frac{\varepsilon_1}{\omega}\sin(\omega t)]}|3,0\rangle. \qquad \text{(A4)}
\end{aligned}$$

For $s_3 = s_4 = 0$ and $s_1, s_2 \neq 0$, Eq. (A4) becomes the SCDT state $|\psi_{0312}(t)\rangle$ in Eq. (19) which describes the Rabi oscillation between the Fock states $|0,3\rangle$ and $|1,2\rangle$, and infers for the SCDT from any one of the states $|0,3\rangle$ and $|1,2\rangle$ to the states $|3,0\rangle$ and $|2,1\rangle$. When $s_1 = s_2 = 0$ and $s_3, s_4 \neq 0$ are set, Eq. (A4) becomes the SCDT state $|\psi_{2130}(t)\rangle$ in Eq. (20). This state means the Rabi oscillation between states $|2,1\rangle$ and $|3,0\rangle$, and the SCDT from any one of states $|2,1\rangle$ and $|3,0\rangle$ to states $|0,3\rangle$ and $|1,2\rangle$.

Case 3: $\eta_3 = 0$.

This case means $E_{1,2} = 0$, $E_{3,4} = \pm\frac{1}{2}\sqrt{3\eta_1^2 + 4\eta_2^2}$, so Eq. (12) becomes the SCDT state $|\psi_{031221}(t)\rangle$ in Eq. (21). It means the Rabi oscillation among states $|0,3\rangle$, $|1,2\rangle$ and $|2,1\rangle$, and the SCDT from any one of states $|0,3\rangle$, $|1,2\rangle$ and $|2,1\rangle$ to state $|3,0\rangle$.

Case 4: $\eta_1 = \eta_3 = 0$.

If we apply $s_1 = s_2 = 0$ and $s_3, s_4 \neq 0$ to Eq. (A3) of the Appendix, this superposition state of Floquet states becomes the SCDT state $|\psi_{1221}(t)\rangle$ in Eq. (22) which describes the Rabi oscillation between states $|1,2\rangle$ and $|2,1\rangle$, and the SCDT from any one of states $|1,2\rangle$ and $|2,1\rangle$ to states $|0,3\rangle$ and $|3,0\rangle$.

Case 5: $\eta_1 = \eta_3 = 0.302$, $\eta_2 = 1.996$.

In order to obtain a SCDT state which can describe the Rabi oscillation between states $|0,3\rangle$ and $|3,0\rangle$, we numerically get the renormalized coupling constants $\eta_1 = \eta_3 = 0.302$ and $\eta_2 = 1.996$ based on Eqs. (8) and (12). Inserting these constants into Eq. (10) yields $A_{1,2} = B_3 = -B_4 = -C_{3,4} = D_1 = -D_2 \approx -0.701$, $A_{3,4} = B_1 = -B_2 = C_{1,2} = -D_3 = D_4 \approx 0.09$, $E_1 = -E_2 \approx 0.034$, $E_3 = -E_4 \approx 2.029$. If the initial state is taken as $|0,3\rangle$, Eqs. (12) and (11) lead to $s_1 = s_2 \approx -0.701$, $s_3 = s_4 \approx 0.09$, and $P_0(t) \approx 0.484 + 0.484\cos(0.067t) + 0.016\cos(1.996t) + 0.016\cos(2.063t)$, $P_1(t) \approx 0.016 - 0.008\cos(0.067t) + 0.016\cos(1.996t) - 0.016\cos(2.063t) - 0.008\cos(4.059t)$, $P_2(t) \approx 0.016 + 0.008\cos(0.067t) - 0.016\cos(1.996t) - 0.016\cos(2.063t) + 0.008\cos(4.059t)$, $P_3(t) \approx 0.484 - 0.484\cos(0.067t) - 0.016\cos(1.996t) + 0.016\cos(2.063t)$.

Obviously, the probabilities obey the inequalities

$$0 \leq P_1(t) + P_2(t) \approx 0.032 - 0.032 \cos(2.063t) \leq 0.064,$$

$$0.936 \leq P_0(t) + P_3(t) \approx 0.968 + 0.032 \cos(2.063t) \leq 1 \tag{A5}$$

so that Eq. (12) becomes the approximate SCDT state $|\psi_{0330}(t)\rangle$ in Eq. (23) of the paper.
